# The musician effect: does it persist under degraded pitch conditions of cochlear implant simulations?

**DOI:** 10.3389/fnins.2014.00179

**Published:** 2014-06-30

**Authors:** Christina D. Fuller, John J. Galvin, Bert Maat, Rolien H. Free, Deniz Başkent

**Affiliations:** ^1^Department of Otorhinolaryngology/Head and Neck Surgery, University Medical Center Groningen, University of GroningenGroningen, Netherlands; ^2^Research School of Behavioral and Cognitive Neurosciences, Graduate School of Medical Sciences, University of GroningenGroningen, Netherlands; ^3^Division of Communication and Auditory Neuroscience, House Research InstituteLos Angeles, CA, USA; ^4^Department of Head and Neck Surgery, David Geffen School of Medicine, UCLALos Angeles, CA, USA

**Keywords:** musician effect, music training, cochlear implant, speech perception, emotion identification, music perception, pitch processing

## Abstract

Cochlear implants (CIs) are auditory prostheses that restore hearing via electrical stimulation of the auditory nerve. Compared to normal acoustic hearing, sounds transmitted through the CI are spectro-temporally degraded, causing difficulties in challenging listening tasks such as speech intelligibility in noise and perception of music. In normal hearing (NH), musicians have been shown to better perform than non-musicians in auditory processing and perception, especially for challenging listening tasks. This “musician effect” was attributed to better processing of pitch cues, as well as better overall auditory cognitive functioning in musicians. Does the musician effect persist when pitch cues are degraded, as it would be in signals transmitted through a CI? To answer this question, NH musicians and non-musicians were tested while listening to unprocessed signals or to signals processed by an acoustic CI simulation. The task increasingly depended on pitch perception: (1) speech intelligibility (words and sentences) in quiet or in noise, (2) vocal emotion identification, and (3) melodic contour identification (MCI). For speech perception, there was no musician effect with the unprocessed stimuli, and a small musician effect only for word identification in one noise condition, in the CI simulation. For emotion identification, there was a small musician effect for both. For MCI, there was a large musician effect for both. Overall, the effect was stronger as the importance of pitch in the listening task increased. This suggests that the musician effect may be more rooted in pitch perception, rather than in a global advantage in cognitive processing (in which musicians would have performed better in all tasks). The results further suggest that musical training before (and possibly after) implantation might offer some advantage in pitch processing that could partially benefit speech perception, and more strongly emotion and music perception.

## Introduction

In normal hearing (NH), musicians show advantages in auditory processing and perception, especially for challenging listening tasks. Musicians exhibit enhanced decoding of affective human vocal sound (Wong et al., 2007; Musacchia et al., 2008; Strait et al., 2009; Besson et al., 2011), better perception of voice cues, and better perception of pitch cues in both speech (prosody) and music (Schon et al., 2004; Thompson et al., 2004; Chartrand and Belin, 2006). But perhaps more importantly, some transfer of musical training to better speech understanding in noise has also been observed, although evidence for such transfer has been mixed (Parbery-Clark et al., 2009; Kraus and Chandrasekaran, 2010; Ruggles et al., 2014). This “musician effect” might be due to better processing of voice pitch cues that can help to segregate speech from noise (Micheyl et al., 2006; Besson et al., 2007; Oxenham, 2008; Deguchi et al., 2012), suggesting that there may be differences between musicians and non-musicians in terms of sound processing at lower levels of the auditory system. Alternatively, the musician effect may be due to better functioning of higher-level processes, such as better use of auditory working memory and attention (Bialystok and DePape, 2009; Besson et al., 2011; Moreno et al., 2011; Barrett et al., 2013).

Previously, the musician effect has been studied in NH listeners under conditions in which the spectro-temporal fine structure cues important for complex pitch perception are fully available. It is not yet known if this effect would persist when the acoustic signal is degraded and when the pitch cues are less available, whether due to signal processing and transmission in hearing devices or by hearing impairment. Such is the case with the cochlear implant (CI), the auditory prosthesis for deaf individuals who cannot benefit from traditional hearing aids. Instead of amplifying acoustic sounds, CIs directly stimulate auditory neurons via electrodes placed inside the cochlea. While the CI users can understand speech transmitted through the device to some degree, this speech signal is greatly reduced in spectral resolution and spectro-temporal fine structure. Further, other factors related to electrode-neuron interface may additionally limit CI performance, such as nerve survival patterns (e.g., Başkent and Shannon, 2006; Bierer, 2010) or potential mismatch in the frequency-place mapping of electric stimulation, especially due to differing electrode array positions inside the cochlea (e.g., Başkent and Shannon, 2007; Holden et al., 2013). As a result, there is large variation in speech perception abilities of CI users post-implantation (Blamey et al., 2013). Furthermore, difficulty understanding speech in noise or in the presence of competing talkers is common among CI users (Friesen et al., 2001; Stickney et al., 2004). The spectro-temporal degradations also severely limit CI users' pitch perception, which is important for recognizing vocal emotion and voice gender, but also for segregating speech from background noise (Fu et al., 2004; Luo et al., 2007; Oxenham, 2008; Fuller et al., under revision). Problems in pitch processing directly and negatively affect musical pitch and timbre perception, and in turn music perception and appreciation (Gfeller et al., 2002a; McDermott, 2004; Galvin et al., 2007; Heng et al., 2011; Limb and Roy, 2014).

Due to aforementioned benefits of the musician effect on speech and music perception, one can argue that music training before or after implantation can also provide some advantages to CI users. In support of this idea, music experience before and after implantation has been shown to benefit CI users' music perception (Gfeller et al., 2000). Further, explicit music training has been observed to significantly improve melodic contour identification (MCI) (Galvin et al., 2007, 2012), and timbre identification and appraisal (Gfeller et al., 2002a; for a review on music appreciation and training in CI users, see Looi et al., 2012). On a potential connection of music training to speech, however, while some CI studies have shown that better music perception was associated with better speech perception (Gfeller et al., 2007; Won et al., 2010), this connection was not always confirmed by other studies. Fuller et al. (2012) showed that previous musical experience with acoustic hearing did not significantly affect CI users' speech performance after implantation. In that study, as is typical for this patient population, few CI participants were trained musicians before implantation, and many reduced their involvement with music after implantation. It is possible that explicit training after implantation may help postlingually deafened CI users to better associate the degraded pitch patterns via electric hearing to pitch patterns developed during previous acoustic hearing. Alternatively, the spectral degradation with CIs may be so severe that previous music experience provides only limited benefit. Thus, it remains unclear whether the musician effect can persist under conditions of spectro-temporal degradation as experienced by CI users.

Acoustic CI simulations have been widely used to systematically explore signal processing parameters and conditions that may affect real CI users' performance. In a typical CI simulation (e.g., Shannon et al., 1995), the input signal is first divided into a number of frequency analysis bands, then the temporal envelope is extracted from each band and used to modulate a carrier signal (typically band-limited noise or sine-wave), and finally the modulated carrier bands are summed. Parameter manipulations can include the number of spectral channels (to simulate different amounts of spectral resolution), the frequency shift between the analysis and the carrier bands (to simulate different electrode insertions), the envelope filter cut-off frequency (to simulate limits on temporal processing), and the analysis/carrier band filter slopes (to simulate different degrees of channel interaction). CI simulations have also been used to elucidate differences and similarities between acoustic and electric hearing under similar signal processing conditions. Friesen et al. (2001) showed that while NH sentence recognition in noise steadily improved as the number of spectral channels in the acoustic CI simulation increased, real CI performance failed to significantly improve beyond 6–8 channels. Luo et al. (2007) found that temporal envelope cues contributed more strongly to NH listeners' vocal emotion recognition with an acoustic CI simulation than in the real CI case. Kong et al. (2004) showed that NH listeners' familiar melody recognition (without rhythm cues) steadily improved as the number of channels were increased in the CI simulation, while real CI performance remained at chance levels despite having 8–22 channels available in the clinical speech processors.

In the present study, CI simulations were used to differentiate the performance between NH musicians and non-musicians, to identify the effect of long-term musical training, when pitch cues must be extracted from a signal that is spectro-temporally degraded given the limited number of channels. The purpose was two-fold; one, to explore to what degree the musician effect would persist under pitch conditions weakened due to spectro-temporal degradations, and two, to explore if the musician effect could potentially be relevant to CI users, for example, by training them with music pre- or post-implantation to increase hearing performance. To achieve this purpose, we systematically investigated the musician effect in a relatively large group of NH participants in three experiments comprised of various speech and music perception tasks, each of which relied on pitch cues to differing degrees. Varying the importance of the pitch cues across the listening tasks might provide insight into mechanisms associated with the musician effect. Speech intelligibility in quiet and in noise was tested using words and sentences. Voice pitch cues would be expected to contribute little to speech understanding in quiet, and possibly more to speech understanding in noise. Vocal emotion identification was tested with and without normalization of amplitude and duration cues that co-vary with fundamental frequency (F0) contours (Luo et al., 2007; Hubbard and Assmann, 2013). Voice pitch cues would be expected to contribute strongly to vocal emotion identification, especially when amplitude and duration cues are less available. MCI was tested with and without a competing masker, in which the pitch and the timbre of the masker and target contours were varied. Pitch cues would be expected to contribute most strongly to MCI, compared to the other listening tasks. All participants were tested in all tasks while listening to unprocessed stimuli or stimuli processed by an 8-channel acoustic CI simulation, using a typical simulation method based on literature. We had three hypotheses: (1) As a direct result of their musical training, musicians would exhibit better music perception. (2) Based on previous studies that showed a transfer from music training to speech perception, we hypothesized that musicians would better understand speech in noise. (3) Based on previous studies that showed a stronger pitch perception in musicians, we hypothesized that musicians would better identify vocal emotion in speech. We further hypothesized that due to better use of pitch cues and better listening skills, musicians would outperform non-musicians also with the CI simulations. However, if musicians outperformed non-musicians in all tasks, this would indicate overall better functioning of high-level auditory perceptual mechanisms. Alternatively, if the musician effect were stronger for listening tasks that relied more strongly on pitch cues, this would indicate that music training mainly improved lower-level auditory perception.

## Experiment 1: speech intelligibility

### Rationale

In Experiment 1, we conducted two tests to explore the musician effect on speech intelligibility: (1) word identification in quiet and in noise at various signal-to-noise ratios (SNRs), and (2) sentence identification in various types of noise. In the test of word identification, there was no semantic context, but the words were meaningful; in the test of sentence identification, there was strong semantic context. Musician effect had been previously observed for speech recognition in noise, but with speech materials with intact spectro-temporal fine-structure cues (Parbery-Clark et al., 2009; Kraus and Chandrasekaran, 2010). To explore the effect of spectral degradation on speech intelligibility along with the musician effect, NH musicians and non-musicians were tested while listening to unprocessed speech or to an acoustic CI simulation.

### Materials and methods

#### Participants

Twenty-five musicians and non-musicians, matched in age and gender, participated in the study (Table 1). Based on previous studies (Micheyl et al., 2006; Parbery-Clark et al., 2009), the inclusion criteria for “musician” were defined as: (1) having begun musical training before or at the age of 7 years, (2) having 10 years or more musical training (i.e., playing an instrument), and (3) having received musical training within the last 3 years on a regular basis. The inclusion criteria for “non-musician” were defined as: (1) not meeting the musician criteria, and (2) not having received musical training within the 7 years before the study. Table 1 shows significant differences between the two participant groups in the number of years of musical training and the starting age of training, confirming a good partition of participants in terms of their music training. There were two small irregularities in participant selection. One non-musician participant started music training at the age of 6 due to mandatory musical training at preliminary school. Another non-musician participant did have 10 years of irregular musical training, but did not have any musical training in the 7 years before the study. Participants were recruited from University of Groningen and from music schools in the area. Further inclusion criteria for all subjects were having NH (pure tone thresholds better than 20 dB HL at the audiometric test frequencies between 250 and 4000 Hz, and 25 dB HL or better at 8 kHz) and being a native Dutch speaker. Exclusion criteria were neurological disorders, especially dyslexia, psychiatric disorders, or untreated past hearing-related problems.

**Table 1:** **Demographics of the participants**.

	**Musicians**	**Non-musicians**	**Comparison of the two groups (*t-test*)**
Mean age (range)	22.9 years (18–27)	22.4 years (19–28)	*t*_(48)_ = −0.78; *p* = 0.440
Gender	7 males; 18 females	7 males; 18 females	N.A.
Mean years of musical training (range)	14.6 years (10–20)	1.6 years (0–10)	*t*_(48)_ = −15.96; *p* < 0.001
Mean age of the start of musical training (range)	5.8 years (3–7)	9.1 years (6–13)	*t*_(33)_ = 3.26; *p* < 0.001

The Medical Ethical Committee of the University Medical Center Groningen (UMCG) approved the study. Detailed information about the study was given and written informed consent was obtained before participation in the study. A financial reimbursement was provided in line with the guidelines of subject reimbursement of Otorhinolaryngology Department of UMCG.

#### Stimuli

***Word identification***. Stimuli included meaningful, monosyllabic Dutch words in CVC format [e.g., bus (“bus,” in English), vaak (“often”), nieuw (“new”), etc.], taken from the NVA test (Bosman and Smoorenburg, 1995). The corpus contains digital recordings of 12 lists, each of which contains 12 words spoken by a female talker. Steady speech-shaped noise (provided with the database) that matched the long-term spectrum of the recordings was used for tests conducted with background noise.

***Sentence identification***. Stimuli included meaningful and syntactically correct Dutch sentences with rich semantic context (Plomp and Mimpen, 1979). The corpus contains digital recordings of 10 lists, each of which contains 13 sentences spoken by a female talker. Each sentence contains 4–8 words. Sentence identification was measured in three types of noise: (1) Steady speech-shaped noise (provided with the database) that matched the long-term spectrum of the recordings, (2) fluctuating noise, the steady speech-shaped noise additionally modulated by the mean temporal envelope of the sentence recordings, and (3) 6-talker speech babble (taken from ICRA noise signals CD, ver.0.3; Dreschler et al., 2001).

Participants were trained with the CI simulation using a different corpus of sentence materials (Versfeld et al., 2000). The training sentences were also meaningful and syntactically correct Dutch sentences with rich semantic context. However, the training sentences were somewhat more difficult compared to the test sentences. The training corpus contains digital recordings of 39 lists, each of which contains 13 sentences spoken by a female talker. Each sentence contains 4–9 words.

#### CI simulation

**Figure 1:**
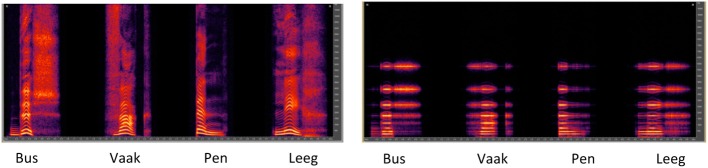
**Spectrograms for Dutch monosyllabic words “Bus,” “Vaak,” “Pen,” and “Leeg” (“Bus,” “Often,” “Pen,” and “Empty” in English), shown for unprocessed speech (left panel) or with the CI simulation (right panel)**.

**Figure 2:**
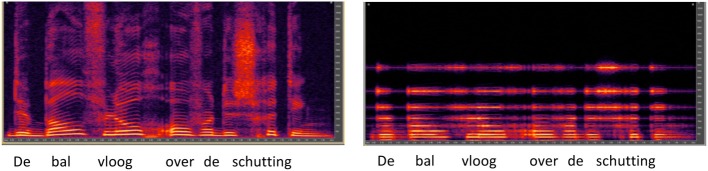
**Spectrograms for Dutch sentence “De bal vloog over de schutting” (“The ball flew over the fence” in English), shown for unprocessed speech (left panel) or with the CI simulation (right panel)**.

An acoustic CI simulation was used to replicate some of the spectral and temporal degradations inherent to CI sound transmission (e.g., Shannon et al., 1995). An 8-channel sinewave vocoder based on the Continuous Interleaved Sampling (CIS) strategy was implemented using Angelsound™ and iStar software (Emily Shannon Fu Foundation, http://angelsound.tigerspeech.com/; http://www.tigerspeech.com/istar). In the simulation, the acoustic input was first band-limited to 200–7000 Hz, which approximates the input frequency range used by many commercial CI devices, and then bandpass-filtered into eight frequency analysis bands (4th order Butterworth filters with band cutoff frequencies according to Greenwood, 1990, frequency-place formula). Eight channels were used in the CI simulation because previous studies have shown that CI users can only access 6–8 spectral channels (e.g., Friesen et al., 2001). For each channel, the temporal envelope was extracted using half-wave rectification and lowpass filtering (4th order Butterworth filter with cutoff frequency = 160 Hz and envelope filter slope = 24 dB/octave). These envelopes were used to modulate a sinusoidal carrier with a frequency that was equal to the center frequency of the analysis filter. The modulated carriers were summed to produce the final stimulus and the overall intensity was adjusted to be the same as the original signal. Figure 1 shows spectrograms for four example Dutch words presented in quiet, for unprocessed speech (left panel) and with the CI simulation (right panel). Similarly, Figure 2 shows spectrograms for an example Dutch sentence presented in quiet, for unprocessed speech (left panel) and with the CI simulation (right panel).

#### Experimental setup

All tests were conducted in an anechoic chamber. Participants were seated in front of a touchscreen (A1 AOD 1908, GPEG International, Woolwich, UK), facing a loudspeaker (Tannoy precision 8D; Tannoy Ltd., North Lanarkshire, UK) at a distance of 1 meter. Stimuli were presented using iStar custom software via a Windows computer with an Asus Virtuoso Audio Device soundcard (ASUSTeK Computer Inc, Fremont, USA). After conversion to an analog signal via a DA10 digital-to-analog converter (Lavry Engineering Inc., Washington, USA) the speech stimulus was played at 65 dBA in free field. The root mean square (RMS) intensity of all stimuli was normalized to the same value. The levels were calibrated with a manikin (KEMAR, GRAS) and a sound-pressure level meter (Type 2610, Brüel Kjær and Sound & Vibration Analyser, Svan 979 from Svantek). Participants' verbal responses on the speech tests were recorded using a DR-100 digital voice recorder (Tascam, California, USA), and were used to double-check responses as needed.

### Procedure

The order of the training and testing sessions was the same for all participants. In each experiment, participants received a short training specific to that experiment. The testing was conducted sequentially in this order: word identification, emotion identification, sentence identification, and MCI. The speech intelligibility data (word and sentence identification) are presented in this section (Experiment 1), the emotion identification data in Experiment 2, and the MCI data in Experiment 3.

#### Training

Participants were trained with the CI simulation and in quiet condition only. Two sentence lists were randomly chosen from the 39 training lists for each participant. The first list was used for passive training, and the second list was used for active training. During passive training, each sentence was played through the loudspeaker and the text was shown simultaneously on the screen. Participants were asked only to listen and to read. After each sentence was presented, the participant pressed “*continue*” on the touchscreen to proceed to the next sentence. After completing the passive training, the touchscreen was turned off. During active training, a training sentence from the second list was played, this time without visual text being displayed. Participants were asked to repeat what they heard as accurately as possible, and to guess if they were unsure of the words. A native Dutch speaker observer, situated in an adjacent room and listening to subjects' responses over headphones, scored the responses using the testing software. Participants were required to score better than 85% correct during active training before beginning formal testing; all participants met this criterion with only one round of active training.

#### Word identification

Word identification was measured with unprocessed speech and the CI simulation in quiet and in steady, speech-shaped noise at 3 SNRs (+10, +5, and 0 dB). One list of 12 words was used to test each condition (eight lists in total). Word lists were randomly chosen from the 12 lists in the test corpus, and no list was repeated for a participant. The order of the conditions was set to progress from relatively easy to relatively difficult: (1) Unprocessed in quiet, (2) CI simulation in quiet, (3) Unprocessed +10 dB SNR, (4) CI simulation +10 dB SNR, (5) Unprocessed +5 dB SNR, (6) CI simulation +5 dB SNR, (7) Unprocessed 0 dB SNR, and (8) CI simulation 0 dB SNR. During testing, a word was randomly selected from within the list and presented via the loudspeaker. The participant was asked to repeat the word as accurately as possible. The observer listened to the responses and scored each correctly repeated phoneme using testing software that calculated the percentage of phonemes correctly recognized. No trial by trial feedback was provided. The total testing time for all conditions was 12–18 min.

#### Sentence identification

Sentence identification was measured with unprocessed speech and the CI simulation in three types of noise: (1) speech-shaped steady noise, (2) speech-shaped fluctuating noise, and (3) 6-talker babble. One list of 13 sentences was used to test each condition (6 lists in total). Sentence lists were randomly chosen from the 10 lists in the test corpus, and no list was repeated for a participant. Similar to word identification testing, the test order for sentence identification was fixed: (1) Unprocessed in steady noise, (2) CI simulation in steady noise, (3) Unprocessed in fluctuating noise, (4) CI simulation in fluctuating noise, (5) Unprocessed in babble noise, and (6) CI simulation in babble noise. For sentence identification in noise, the speech reception threshold (SRT), defined as the SNR needed to produce 50% correct sentence identification, was measured using an adaptive, one-up/one-down procedure (Plomp and Mimpen, 1979), in which the SNR was adjusted from trial to trial according to the accuracy of the response. During testing, speech and noise were presented at the target SNR over the loudspeaker and the participant was asked to repeat the sentence as accurately as possible. If the participant repeated all words in the sentence correctly, the SNR was reduced by 2 dB; if the participant did not repeat all words in the sentence correctly, the SNR was increased by 2 dB. The reversals in SNR between trials 4–13 was averaged and reported as the SRT for the test condition. To better target the SRT within the limited number of sentences in the test list, the initial SNR was different for each noise type and listening condition, based on preliminary testing. For steady noise, the initial SNRs were −4 and +2 dB for unprocessed speech and the CI simulation, respectively. For fluctuating noise, the initial SNRs were −8 and +6 dB for unprocessed speech and the CI simulation, respectively. For babble, the initial SNRs were −4 and +6 dB for unprocessed speech and the CI simulation, respectively. Note that the first sentence was repeated and the SNR increased until the participant repeated the entire sentence correctly. The total testing time for all conditions was 15–20 min.

### Results

#### Word identification

**Figure 3:**
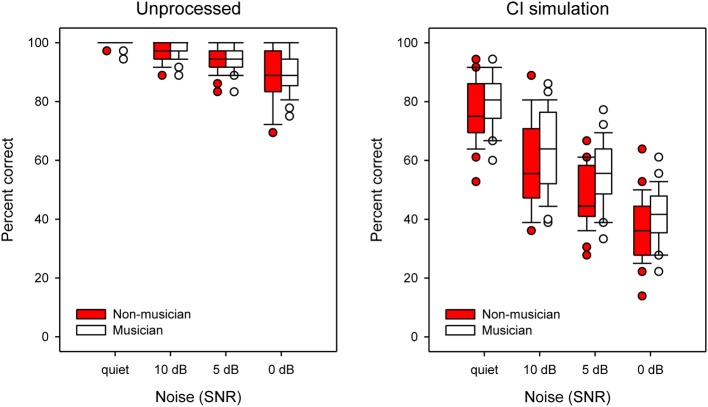
**Boxplots of word identification scores for musicians and non-musicians shown as a function of SNR**. The left and right panels show data with unprocessed stimuli or with the CI simulation, respectively. The error bars show the 10 and 90th percentiles and the circles show outliers.

Figure 3 shows boxplots for word identification performance by musicians (white boxes) and non-musicians (red boxes) listening to unprocessed stimuli (left panel) or the CI simulation (right panel), as a function of noise condition. Performance generally worsened as the noise level increased, for both listening conditions, and performance with the CI simulation was generally poorer than that with unprocessed stimuli. In the CI simulation, musicians generally performed better than non-musicians. A split-plot repeated measures analysis of variance (RM ANOVA) was performed on the data, with group (musician, non-musician) as the between-subject factor, and listening condition (unprocessed, CI simulation) and SNR (quiet, +10, +5, and 0 dB) as within-subject factors. The complete analysis (with Greenhouser-Geisser corrections due to sphericity violations) is presented in Table 2. There were significant main effects for subject group [*F*_(1, 48)_ = 7.76; *p* = 0.008], listening condition [*F*_(1, 48)_ = 1098.55; *p* < 0.001] and SNR [*F*_(2.63, 126.36)_ = 409.85; *p* < 0.001]. There was a significant interaction between listening condition and SNR [*F*_(2.81, 134.67)_ = 148.54; *p* < 0.001]. Despite the overall main group effect, *post-hoc t-tests* showed a significant difference between musicians and non-musicians only at the +5 dB SNR with CI simulation [*t* = −2.94; *df* = 48; *p* = 0.005], namely, at one condition out of eight tested.

**Table 2:** **Experiment 1: Results of a split-plot RM ANOVA (with Greenhouse-Geisser correction) for word identification**.

		**Observed power**
**BETWEEN-SUBJECT FACTOR**		
Group	*F*_(1, 48)_ = 7.76; *p* = 0.008^*^	0.78
**WITHIN-SUBJECT FACTORS**		
Listening condition	*F*_(1, 48)_ = 1098.55; *p* < 0.001^*^	1.00
SNR	*F*_(2.63, 126.36)_ = 409.85; *p* < 0.001^*^	1.00
SNR × listening condition	*F*_(2.81, 134.67)_ = 148.54; *p* < 0.001^*^	1.00
SNR × group	*F*_(2.63, 126.36)_ = 2.02; *p* = 0.449	0.20
Listening condition × group	*F*_(1, 48)_ = 1.02; *p* = 0.051	0.50
Listening condition × SNR × group	*F*_(2.81, 134.67)_ = 0.95; *p* = 0.416	0.25

* *Significant (p < 0.05)*.

#### Sentence identification

**Figure 4:**
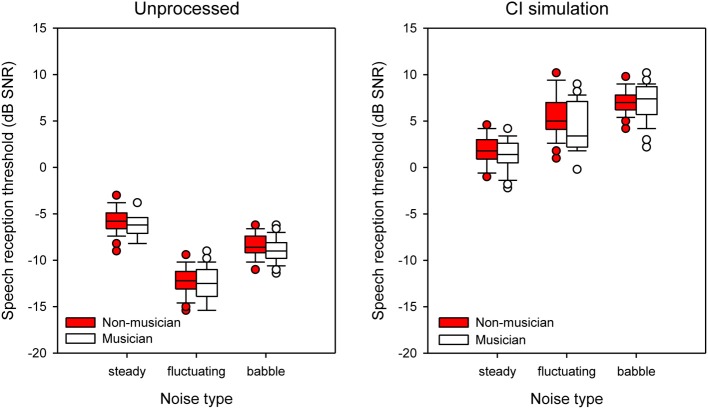
**Boxplots of SRTs for musicians and non-musicians shown as function of different noise types for different noise conditions**. The left and right panels show data with unprocessed stimuli or with the CI simulation, respectively. The error bars show the 10 and 90th percentiles and the circles show outliers.

Figure 4 shows boxplots for SRTs by musicians (white boxes) and non-musicians (red boxes) listening to unprocessed stimuli (left panel) or the CI simulation (right panel), as a function of noise type. With unprocessed speech, performance was generally best with the fluctuating noise and poorest with the steady noise. With the CI simulation, performance was generally best with steady noise and poorest with babble. Performance with unprocessed speech was much better than with the CI simulation. Differences between musicians and non-musicians were generally small. A split-plot RM ANOVA was performed on the data, with group as the between-subject factor, and listening condition and noise type (steady, fluctuating, babble) as within-subject factors. The complete analysis is presented in Table 3. There were significant main effects for listening condition [*F*_(1, 48)_ = 3771.1; *p* < 0.001] and noise type [*F*_(1.56, 74.97)_ = 95.01; *p* < 0.001], but not for group [*F*_(1, 48)_ = 2.85; *p* = 0.098]; note that the observed power was relatively weak for the group comparison (0.38). There was a significant interaction between listening condition and noise type [*F*_(1.80, 86.54)_ = 273.90; *p* < 0.001]. *Post-hoc* tests did not show any significant differences between groups with the different noise types.

**Table 3:** **Experiment 1: Results of a split-plot ANOVA (with Greenhouse-Geisser correction) for sentence identification**.

		**Observed power**
**BETWEEN-SUBJECT FACTOR**		
Group	*F*_(1, 48)_ = 2.85; *p* = 0.098	0.38
**WITHIN-SUBJECT FACTORS**		
Listening condition	*F*_(1, 48)_ = 3771.1; *p* < 0.001^*^	1.00
Noise type	*F*_(1.56, 74.97)_ = 95.01; *p* < 0.001^*^	1.00
Noise type × listening condition	*F*_(1.80, 86.54)_ = 273.90; *p* < 0.001^*^	1.00
Noise type × group	*F*_(1.56, 74.97)_ = 0.46; *p* = 0.587	0.11
Listening condition × group	*F*_(1, 48)_ = 0.17; *p* = 0.682	0.07
Listening condition × noise type × group	*F*_(1.80, 86.54)_ = 1.05; *p* = 0.350	0.22

* *Significant (p < 0.05)*.

## Experiment 2: identification of emotion in speech

### Rationale

In Experiment 2, a vocal emotion identification task was used to test whether there was a musician effect for a speech-related test that heavily relied on perception of pitch cues in speech. To avoid any influence of semantic content on performance, a nonsense word was used to produce the target emotions. Although pitch cues strongly contribute to emotion identification, other cues such as duration and amplitude co-vary with pitch and can also be used for this purpose (Luo et al., 2007; Hubbard and Assmann, 2013). Accordingly, vocal emotion identification was tested for speech stimuli in two versions; once with pitch, duration and amplitude cues preserved across stimuli, and once with duration and amplitude cues normalized across stimuli, leaving in mainly the pitch cues. When duration and amplitude cues are minimal, vocal emotion identification is more difficult, especially under conditions of CI signal processing in which pitch cues are also weakened (Luo et al., 2007). Testing with normalized stimuli would thus allow performance to be compared between musicians and non-musicians when mainly pitch cues are available, with other acoustic cues minimized.

As in Experiment 1, musicians and non-musicians were tested while listening to unprocessed stimuli or to a CI simulation. Participants, CI simulation, and general experimental setup were identical to Experiment 1. The differences in design are explained below.

### Stimuli

Stimuli included digital recordings made by Goudbeek and Broersma (2010). The original corpus contains a nonsense word [nutohɔmsεpikɑη] produced by eight professional Dutch actors according to eight target emotions. The actors, who were all trained or were in training at a drama school, were instructed to imagine emotions in a scenario or by reliving personal episodes in which the target emotion occurred. Based on a pilot study with three participants, the four actors (two female, two male), and the four emotions representing all corners of the emotion matrix were chosen for formal testing (Goudbeek and Broersma, 2010). Target emotions included: (1) Anger (high arousal, negative valence), (2) Sadness (low arousal, negative valence), (3) Joy (high arousal, positive valence), and (4) Relief (low arousal, positive valence). This resulted in a total of 32 tokens (4 speakers × 4 emotions × 2 utterances).

**Figure 5:**
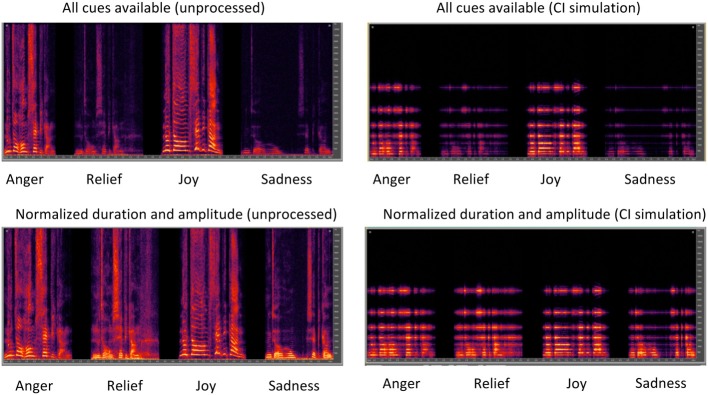
**Spectrograms for Dutch nonsense words produced according to four target emotions**. The left panels show unprocessed speech and the right panels show speech processed with the CI simulation. The top panels show speech with duration, amplitude, and pitch cues intact. The bottom panels show speech with normalized duration and amplitude cues, but with preserved pitch cues.

For the intact stimuli, duration ranged 1.06–2.76 s and amplitude ranged 45–80 dBA. For the normalized stimuli, duration was normalized to 1.77 s using a script in PRAAT (version 5.3.16; Boersma and Weenink, 2012) without changing the fundamental frequency, and amplitude normalized to 65 dBA using Matlab (i.e., the mean duration and amplitude of the intact stimuli). Figure 5 shows spectrograms for the four target emotions with all cues intact (top panels) or with normalized duration and amplitude cues (bottom panels); the left panels show unprocessed speech and the right panels show speech processed with the CI simulation.

### Procedure

For all participants, conditions were tested in a fixed order: (1) Original (with all cues intact), unprocessed stimuli, (2) Original, CI simulation, (3) Normalized (in duration and amplitude), unprocessed, and (4) Normalized, CI simulation. Stimuli were presented using Angelsound software™. Before formal testing, participants were familiarized with the test procedure while listening to unprocessed stimuli, namely, the target emotions (intact stimuli only) produced by four actors not used for formal testing. During training, a target emotion was randomly selected from the stimulus set and presented over the loudspeaker. Subjects were asked to indicate the emotion of the stimulus by touching one of four response boxes on the touchscreen labeled “anger,” “sadness,” “joy,” and “relief.” Visual feedback was provided on the screen, and in case of an incorrect answer, the correct response and incorrect response were replayed. The actual data collection was identical to training, except that no audio-visual feedback was provided and only the selected test stimuli were used. The software calculated the percent correct and generated confusion matrices. The total testing time for all conditions was 8–16 min.

### Results

**Figure 6:**
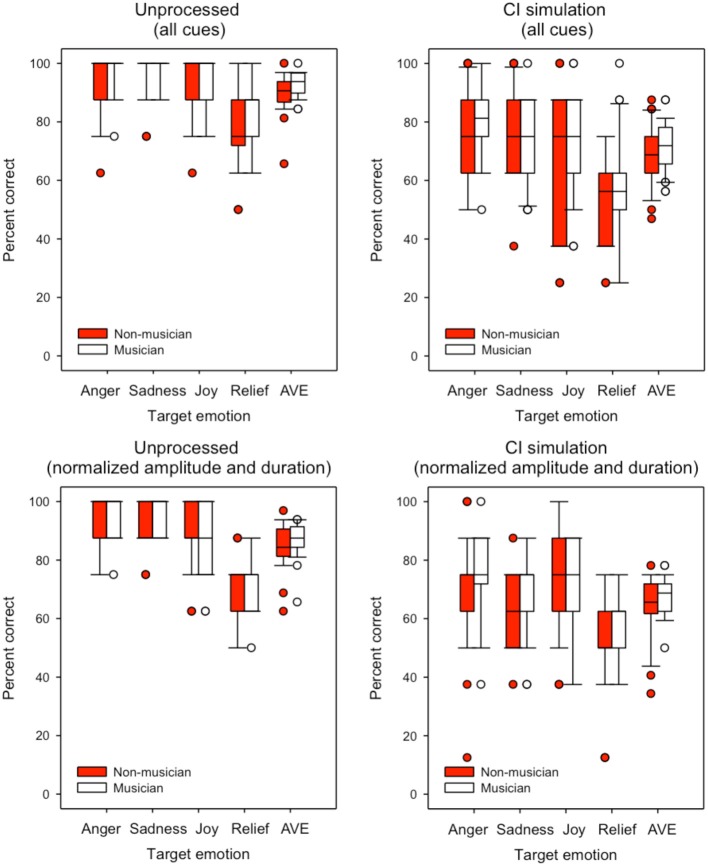
**Boxplots for identification of each emotion and overall emotion identification for musicians and non-musicians**. The left and right panels show data with unprocessed stimuli or with the CI simulation, respectively. The top panels show performance with pitch, duration, and amplitude cues preserved and the bottom panels show performance with normalized duration and amplitude cues. The error bars show the 10 and 90th percentiles and the circles show outliers.

Figure 6 shows boxplots for emotion identification by musicians (white boxes) and non-musicians (red boxes) listening to unprocessed stimuli (left panels) or the CI simulation (right panels); the top panels show performance with pitch, duration, and amplitude cues preserved and the bottom panels show performance with normalized duration and amplitude cues. Note that in some cases, median and 25th/75th percentiles could not be displayed because performance was similarly good amongst participants; as such, only error bars and outliers are displayed. In general, “relief” was the least reliably recognized emotion. Performance generally worsened when duration and amplitude cues were normalized. There was a small advantage for musicians in all test conditions. A split-plot RM ANOVA was performed on the data, with group as the between-subject factor, and listening condition and cue availability (all cues, normalized duration and amplitude) as within-subject factors. The complete analysis is presented in Table 4. There were significant main effects for group [*F*_(1, 48)_ = 4.66; *p* = 0.036], listening condition [*F*_(1, 48)_ = 323.85; *p* < 0.001] and cue availability [*F*_(1, 48)_ = 18.59; *p* < 0.001].

**Table 4:** **Experiment 2: Results of split-plot RM ANOVA for emotion identification**.

		**Observed power**
**BETWEEN-SUBJECT FACTOR**		
Group	*F*_(1, 48)_ = 4.66; *p* = 0.036^*^	0.56
**WITHIN-SUBJECT FACTORS**		
Cue availability	*F*_(1, 48)_ = 18.59; *p* < 0.001^*^	0.99
Listening condition	*F*_(1, 48)_ = 323.85; *p* < 0.001^*^	1.00
Cue availability × group	*F*_(1, 48)_ = 0.12; *p* = 0.733	0.06
Listening condition × group	*F*_(1, 48)_ = 0.21; *p* = 0.648	0.07
Cue availability × listening condition	*F*_(1, 48)_ = 1.19; *p* = 0.281	0.19
Cue availability × listening condition × group	*F*_(1, 48)_ = 0.03; *p* = 0.863	0.05

* *Significant (p < 0.05)*.

## Experiment 3: melodic contour identification

### Rationale

In Experiment 3, a MCI (Galvin et al., 2007) task was used to test musicians' and non-musicians' perception of musical pitch and ability to use timbre and pitch cues to segregate competing melodies. Participants were asked to identify a target melodic contour from among a closed-set of responses that represented various changes in pitch direction. MCI was measured for the target alone, and in the presence of a competing contour. The timbre of the target contour and the pitch of the competing contour were varied to allow for different degrees of difficulty in segregating the competing contours. As in Experiments 1 and 2, participants were tested while listening to unprocessed stimuli or the CI simulation. The degradations imposed by CI simulation were expected to have a profound effect on MCI performance, given that melodic pitch was the only cue of interest and would not be well represented in the CI simulation. As this experiment was a more direct measure of music perception, musicians were expected to perform better than non-musicians.

Participants, CI simulation, and general experimental setup were identical to Experiments 1 and 2. Details of the experimental stimuli and procedures are described below.

### Stimuli

**Figure 7:**
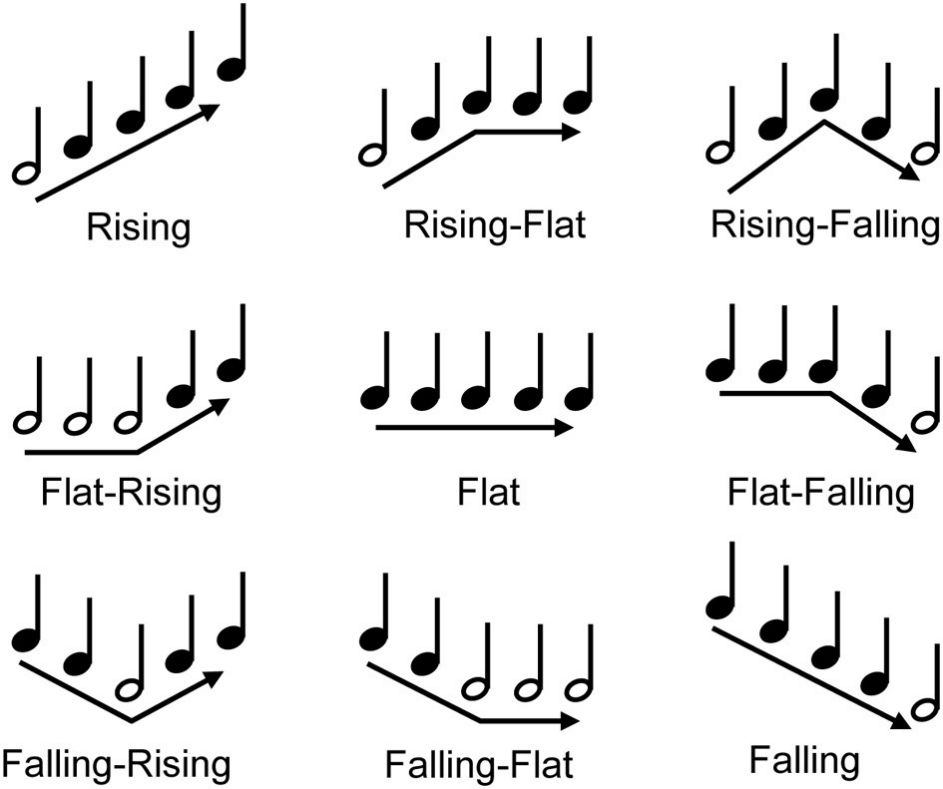
**The nine melodic contours used for MCI testing**. The white note shows the lowest note of the contour (A3; 220 Hz).

**Figure 8:**
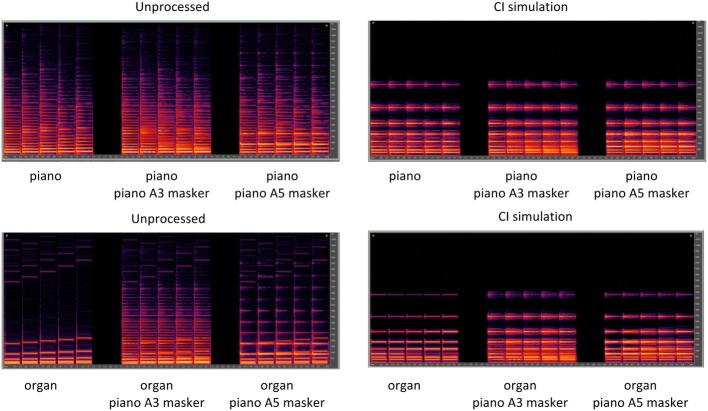
**Spectrograms for a Rising target melodic contour with 1-semitone spacing**. The top panels show the piano target with the piano A3 and A5 maskers and the bottom panels show the organ target with the piano A3 and A5 maskers. The left panels show unprocessed signals and the right panels show signals processed by the CI simulation.

Stimuli for the MCI test consisted of nine 5-note melodic contours (see Figure 7) that represented different changes in pitch direction: “Rising,” “Flat,” “Falling,” “Flat-Rising,” “Falling-Rising,” “Rising-Flat,” “Falling-Flat,” “Rising-Falling,” “Flat-Falling.” The lowest note in a given contour was A3 (220 Hz). The spacing between successive notes in the contour was 1, 2, or 3 semitones. Presumably, the 1 semitone spacing would be more difficult than the 3 semitone spacing, as the contours would be represented by a smaller cochlear extent. The duration of each note was 250 ms, and the silent interval between notes was 50 ms. The target contour was played by either a piano or an organ sample, as in Galvin et al. (2008). MCI was measured for the target alone or in the presence of a competing contour, as in Galvin et al. (2009). The competing contour (“masker”) was always the “Flat” contour, played by piano sample. The pitch of the masker was varied to overlap the pitch of the target, or not. The overlapping pitch was A3 (220 Hz); the non-overlapping pitch was A5 (880 Hz). Thus, there were six conditions: (1) piano target alone (no masker), (2) piano target with the A3 piano masker, (3) piano target with the A5 piano masker, (4) organ target alone (no masker) (5) organ target with the A3 piano masker, and (6) organ target with the A5 piano masker. It was expected that MCI performance would be best with no masker, better with the organ than the piano, and better with the A5 than the A3 masker. As such, performance with the organ target with the A5 piano masker (i.e., maximum pitch and timbre difference) would be expected to be better than that with the piano target with the A3 piano masker (minimum pitch and timbre difference). The masker onset and offset was identical to the target contour; thus the notes of the masker and the target occurred simultaneously. Figure 8 shows spectrograms for the Rising target contour played either by the piano (top panels) or the organ (bottom panels). In each panel, the target contour is shown, from left to right, with no masker, with the overlapping A3 piano masker, and with the non-overlapping A5 piano masker.

### Procedure

MCI testing procedures were similar to previous studies (Galvin et al., 2007, 2008, 2009). Before formal testing, participants were trained in the MCI procedure. The piano and organ samples were used for training; only the target contours were presented. During training for both piano and organ (both normal and CI simulated stimuli), a contour was randomly selected and presented via the loudspeaker. The participant was instructed to pick the contour that best matched the stimulus from among nine response choices shown on the screen; the response boxes were labeled with both a text descriptor (e.g., “Rising,” Falling,” Flat,” etc.) and an illustration of the contour. After responding, visual feedback was provided and in the case of an incorrect response, audio feedback was provided in which the correct response and the participant's (incorrect) response were played in sequence.

Testing methods were the same as for the training, except that no feedback was provided. For all participants, the test order was fixed: (1) piano target (no masker), unprocessed, (2) piano target (no masker), CI simulation, (3) piano target with piano A3 masker, unprocessed, (4) piano target with piano A3 masker, CI simulation, (5) piano target with piano A5 masker, unprocessed, (6) piano target with piano A5 masker, CI simulation, (7) organ target (no masker), unprocessed, (8) organ target (no masker), CI simulation, (9) organ target with piano A3 masker, unprocessed, (10) organ target with piano A3 masker, CI simulation, (11) organ target with piano A5 masker, unprocessed, and (12) organ target with piano A5 masker, CI simulation. For conditions with a masker, participants were instructed that the masker would always be the “Flat” contour (i.e., the same note played five times in a row), and to ignore the masker and listen for the target, which would change in pitch. Responses were recorded using the test software, and the percent correct was calculated for each condition. The total testing time for all conditions was approximately 30 min.

### Results

**Figure 9:**
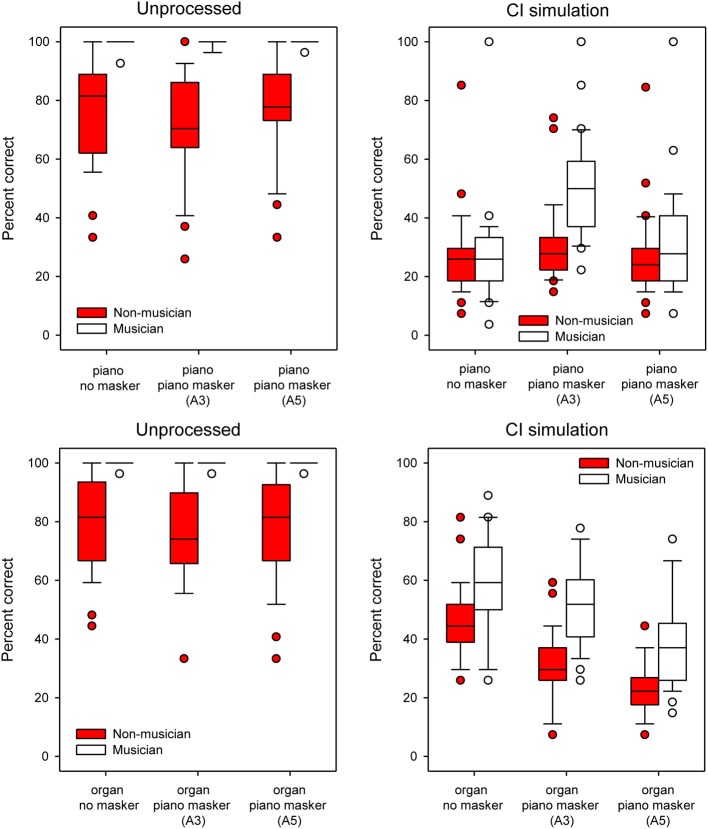
**Boxplots for MCI performance for each masker condition for musicians and non-musicians**. The top and bottom panels show data for the piano and organ targets, respectively. The left and right panels show data with unprocessed stimuli or with the CI simulation, respectively. Within each panel, data is shown with no masker, with the A3 piano masker, and with the A5 piano masker. The error bars show the 10th and 90th percentiles and the circles show outliers.

Figure 9 shows box plots of MCI performance with unprocessed stimuli (left panel) or with the CI simulation (right panel), for musicians (white boxes) and non-musicians (red boxes), as a function of test condition. Note that in some cases, median and 25th/75th percentiles could not be displayed because performance was similarly good amongst participants; as such, only error bars and outliers are displayed. In general, musicians outperformed non-musicians; with unprocessed signals, musician performance was nearly perfect, even with the competing masker. Performance for both groups was much poorer with the CI simulation. The effects of the masker were unclear and somewhat counter-intuitive. In the CI simulation, performance was generally better with the A3 than with the A5 maskers, suggesting that listeners could not make use of the pitch difference between the target and the masker. Similarly, the effects of timbre were small in the CI simulation, as performance was generally similar between the piano and the organ. A split-plot RM ANOVA was performed on the data, with group as the between-subject factor, and target timbre (piano and organ) and masker pitch (no masker A3, A5) as within-subject factors. The complete analysis is presented in Table 5. There were significant main effects for group [*F*_(1, 48)_ = 59.52; *p* < 0.001], target timbre [*F*_(1, 48)_ = 69.60; *p* < 0.001], listening condition [*F*_(1, 48)_ = 993.84; *p* < 0.001], and masker pitch [*F*_(1.85, 88.71)_ = 14.66; *p* < 0.001]. *Post-hoc t-tests* showed a significant effect of group for all conditions for the unprocessed stimuli (*p* < 0.001). For the CI simulation, a significant musician effect was shown for the piano target with the piano A3 masker [*t*_(48)_ = −5.10, *p* < 0.001], the organ target with no masker [*t*_(48)_ = −2.89, *p* = 0.006], the organ target with the piano A3 masker [*t*_(48)_ = −5.52, *p* < 0.001] and the organ target with the piano A5 masker [*t*_(48)_ = −4.22, *p* < 0.001].

**Table 5:** **Experiment 3: Results from a split-plot RM ANOVA for melodic contour identification**.

		**Observed power**
**BETWEEN SUBJECT FACTOR**		
Group	*F*_(1, 48)_ = 59.52; *p* < 0.001^*^	1.00
**WITHIN SUBJECT FACTORS**		
Target timbre	*F*_(1, 48)_ = 69.60; *p* < 0.001^*^	1.00
CI simulation	*F*_(1, 48)_ = 993.84; *p* < 0.001^*^	1.00
Masker pitch	*F*_(1.85, 88.71)_ = 14.66; *p* < 0.001^*^	1.00
Target timbre × masker pitch	*F*_(1.76, 84.69)_ = 56.67; *p* < 0.001^*^	1.00
Target timbre × CI simulation	*F*_(1, 48)_ = 55.55; *p* < 0.001^*^	1.00
Target timbre × group	*F*_(1, 48)_ = 2.90; *p* = 0.095	0.39
CI simulation × group	*F*_(1, 48)_ = 11.19; *p* = 0.002^*^	0.91
CI simulation × masker pitch	*F*_(1.96, 93.82)_ = 27.51; *p* < 0.001^*^	1.00
Masker pitch × group	*F*_(1.85, 88.71)_ = 10.45; *p* < 0.001^*^	0.98
Target timbre × CI simulation × Group	*F*_(1, 48)_ = 19.86; *p* < 0.001^*^	0.99
Target timbre × CI simulation × Masker	*F*_(1.95, 93.62)_ = 46.22; *p* < 0.001^*^	0.99
Target timbre × masker pitch × group	*F*_(1.76, 84.69)_ = 3.21; *p* = 0.051	0.56
Target timbre × CI simulation × masker pitch × group	*F*_(1.95, 93.62)_ = 1.56; *p* = 0.217	0.32

* *Significant (p < 0.05)*.

## General discussion

The study showed an overall musician effect, however, the degree of the musician effect varied greatly across the three experiments. The musician effect was largest for the music test, even with melody contours degraded through a CI simulation, most likely as a direct consequence of music training. The musician effect was smaller for emotion identification, which relied strongly on perception of voice pitch contours, especially for the normalized stimuli where other potential cues, such as intensity and duration, were minimized; however, musicians still outperformed non-musicians even after the pitch cues were also degraded through the CI simulation. For speech perception, there was limited musician effect observed with only one of the speech tests used, word identification, and then only for one out of eight conditions tested, with the CI simulation and presented with background noise at +5 dB SNR.

### The musician effect

As outlined in the Introduction, there are two plausible explanations for why musicians may perceive speech better. First, musicians may be better able to detect pitch cues in stimuli, allowing for better segregation of acoustic cues that may improve speech intelligibility in challenging situations (Micheyl et al., 2006; Besson et al., 2007; Oxenham, 2008; Deguchi et al., 2012). Second, musicians may be better overall listeners due to better high-level auditory cognitive functioning, such as in working memory and auditory attention (Bialystok and DePape, 2009; Besson et al., 2011; Moreno et al., 2011; Barrett et al., 2013), which can also improve speech intelligibility, not only in noise (Parbery-Clark et al., 2009), but also in general. The present data suggest that better pitch processing more strongly contributed to the musician effect, at least for the specific sets of experiments employed. This observation is in line with literature that has shown musicians to rely more heavily on pitch cues than non-musicians when stimuli are degraded (e.g., Fuller et al., 2014). Further, musicians seem to have a better pitch percept in pitch-related tasks in both speech and music, shown not only behaviorally, but also in imaging studies with an enhanced processing at different brain levels (Besson et al., 2011). Because it was not explicitly tested in this study, how higher-level cognitive processing may have contributed to the present pattern of results is difficult to judge. However, the observation that the musician effect increased as pitch cues became more meaningful across listening tasks suggests that pitch perception was a strong factor that differentiated musicians from non-musicians.

Prior evidence for transfer of music training to speech perception has been mixed. While Parbery-Clark et al. (2009) showed a small musician effect for identification of sentences presented in noise, but not processed otherwise, Ruggles et al. (2014) showed no musician effect for identification of sentences in noise, presented with or without voice pitch cues. In the present study, there was a significant musician effect for word identification (Experiment 1), yet, this was limited to one condition out of eight tested, only observed in noise and with CI simulation, and there was no musician effect for sentence recognition in noise, with or without CI simulation. The reason for not observing an effect in the latter may be that sentence recognition depends on also other factors besides pitch perception (e.g., segregating speech from noise, extracting meaning with help from semantics, context, prosody, and also using higher-level cognitive and linguistic processes). If the musician effect is largely based on pitch processing, it may be more difficult to observe with sentences; this effect may be stronger when perceiving subtle speech cues in phonetics-based tasks such as identification of syllables (Zuk et al., 2013) or words (in the present study), but this effect may diminish for linguistically rich materials, such as sentences, where listeners can compensate degradations using linguistic skills as well (Benard et al., 2014). Hence, overall, the present data combined with past studies imply that there could be some transfer of music training to better perception of speech, especially in degraded listening conditions, but this effect seems to be rather small. Further, this is perhaps a consequence of the musician advantage being mainly due to better processing of low-level acoustic cues, instead of a better overall cognitive processing.

The musician effect may be stronger in speech-related tasks in which pitch cues are more important. After all, perception of speech prosody is vital to real-life speech communication and depends strongly on perception of pitch cues (Wennerstrom, 2001; Besson et al., 2011). One novel aspect of the present study was to include the emotion identification task to explore this idea (Experiment 2). In this test, musicians were expected to have an advantage due to better utilization of pitch cues, as in comparison to neutral speech, angry and happy speech exhibit a wider pitch range as well as a higher mean pitch, while sad speech has a narrower range and lower mean pitch (Banse and Scherer, 1996; Luo et al., 2007). In line with this idea, Globerson et al. (2013) had observed that listeners with better F0 identification also exhibited better emotion identification in speech. However, other acoustic cues also contribute to vocal emotion identification, such as the level and the range of the duration and amplitude (controlled for in the present study), but also vocal energy, tempo, and pausing (not controlled; Hubbard and Assmann, 2013); hence, it was not known before the present study if musician advantage indeed would also present an advantage in perception of vocal emotion in speech. In the present study, we measured emotion identification in a nonsense word (thereby removing any semantic cues) in two versions; once with all cues intact, and once with normalized duration and amplitude cues, leaving mainly the pitch cues intact. There was a small but significant overall group effect, with no interactions with presence or absence of CI simulations or of normalization of other cues than pitch, confirming that generally musicians perceived vocal emotion in speech better than non-musicians. Consistent with previous literature (Thompson et al., 2004; Besson et al., 2007), the present data suggest that musicians may better utilize the pitch cues for vocal emotion identification, but interestingly, this is a persistent effect as they do so even when pitch cues are degraded through a CI simulation.

Note that, although twenty-five musicians and non-musicians were recruited based on a power-analysis prior to the study, the observed power for some analyses was low. This could either mean that there were not enough participants and/or that the musician effect was too small. For example, the observed power for the sentence test in stationary noise was 0.38 (Table 3). A power analysis based on the present results indicated that there would need to be a very large number of participants to achieve adequate power. Therefore, a musician effect for this specific test would not likely be found by increasing the number of participants in a realistic manner, and such a small effect might not be relevant in daily life. On the other hand, the observed power for the emotion test was 0.56 (Table 4), and while low, this was sufficient to produce statistically significant effects. For this test, to achieve power = 0.80, the number of participants would need to be increased to 46. As such, for this test, further research with more participants has the potential to produce more significant differences between musicians and non-musicians.

### Effect of the CI simulation

For all test conditions, mean performance was poorer with the CI simulation than with unprocessed speech, for both musicians and non-musicians. The effect of the CI simulation was more pronounced for more difficult listening tasks (e.g., speech recognition in noise, MCI). The musician effect persisted (or appeared, in the case of speech perception) with the application of the CI simulation, hinting that musicians were better able to extract acoustic cues in degraded conditions than non-musicians.

Interestingly, the effect of different types of noise also varied between unprocessed and CI-simulated conditions. In NH, a release of masking is observed when same listeners are tested with a steady noise vs. a fluctuating noise, usually resulting in better speech perception performance with the latter (Miller and Licklider, 1950; Başkent et al., 2014). This improvement is usually attributed to the glimpses of speech available through the valleys, i.e., low-level portions of the fluctuating noise, which provide samples of the speech that the listener can make use of to restore speech for enhanced intelligibility. In the present study, while there was such release from masking for unprocessed speech with fluctuating maskers, performance worsened with fluctuating maskers for the CI simulation. Such effects of dynamic maskers have been previously observed with real CI users and in CI simulations (Nelson et al., 2003; Fu and Nogaki, 2005). The limited spectral resolution, due to both the limited number of channels and the interactions between channels, is thought to increase susceptibility to fluctuating maskers in both CI users and CI simulations. Further, recent work by Bhargava et al. (2014) showed that perhaps the reduced quality of the speech glimpses due to signal degradations in CIs make them also more difficult to utilize the top-down reconstruction of speech in fluctuating noise. These factors can also limit melodic pitch perception in CI simulations. For example, Crew et al. (2012) showed that, even when the number of channels was increased, MCI performance was quite poor when there was substantial channel interaction in the CI simulations. Most likely, the current spread across electrodes in real CIs similarly causes spectral smearing, reducing the functional spectral resolution to be less than the number of nominal channels, thereby limiting the release from masking, as well as pitch perception.

Note that sinewave vocoding was used for the present CI simulation, rather than noise-band vocoding. The sinewave vocoder was used because of the greater specificity in terms of place of cochlear stimulation, as well as better representation of the temporal envelope, which may be “noisier” with noise-band carriers (e.g., Fu et al., 2004). One potential problem with sinewave vocoding, however, is the introduction of side-bands around the carrier frequency. Such side-band information would not be available in the case of real CIs. Although these side-bands may have provided additional (albeit weak) spectral cues beyond the eight sinewave carriers, these cues would have been available to both musicians and non-musicians in this study. It may be that musicians were better able to use this side-band information, or were better able to use pitch cues encoded in the temporal envelope. Either way, musicians in general performed better than non-musicians in the CI simulation. This observation gives support to previous literature (Gfeller et al., 2000; Galvin et al., 2007, 2012; Looi et al., 2012), and implies that musically trained CI users might be better able to perceive much-weakened pitch cues delivered by their devices (e.g., Fuller et al., 2014, under revision).

### Implications for cochlear implant users

The patterns of musician effect observed with unprocessed stimuli did not change largely with the CI simulations, except for generally poorer performance, and in case of speech intelligibility, the musician effect only appeared after the CI simulation was applied. This implies that the musician effect seems to persist despite the signal degradations associated with CI signal processing, or may become even more important in the presence of such degradations where listeners can benefit even more greatly if they can perceive any acoustic cues, albeit weak. While this sounds promising, one has to be cautious before drawing strong conclusions regarding actual CI users, whose demographics vary from that of young NH populations, and who also have to deal with additional factors related to the device front-end processing and nerve-electrode interface. One important consideration is that most post-lingually deafened CI users are typically older than the present study participants (Blamey et al., 2013), and have experienced a period of auditory deprivation (Lazard et al., 2012). Age alone can alter the cognitive and linguistic processes needed for speech perception in noise (e.g., Başkent et al., 2014), and auditory deprivation may lead to structural changes in the brain, affecting overall sound perception (e.g., Lazard et al., 2014). Thus, the sometimes small musician effects in this study, measured under ideal and well-controlled conditions, may be even smaller in actual CI users. Alternatively, to their benefit, real CI users will have had much greater experience with the CI signal processing than the NH participants of the present study had experience with the simulated CI. As the actual users of CIs have to rely on these degraded signals exclusively, and will have (had) more time to practice with them, the small effects observed in this study may have greater consequences for actual CI users' real-life performance.

Previous studies have shown significant benefits of musical training after implantation for post-lingually deafened CI users' music perception (Gfeller et al., 2000, 2002b; Galvin et al., 2007, 2012; Driscoll et al., 2009). In the present study, musical training, the main factor that differentiated the musician group from the non-musician group, was associated with better performance as pitch cues became more important in the listening task. Training melodic pitch perception in CI users may also benefit music perception and speech perception where pitch cues are relevant (emotion recognition, prosody perception, segregation of speech from background noise or distractor signals, etc.). However, such training will likely differ from the long-term music training experienced by the present group of NH musicians. Learning to play an instrument, with spectro-temporal fine-structure cues available and over a period of many years, may give rise to robust central pitch representations. Training melodic pitch perception after implantation may not provide such robust patterns. On the other hand, an earlier training provided to hearing-impaired children before they reach the level of profound hearing loss may provide positive results, due to yet strong plasticity experienced in childhood (Hyde et al., 2009; Moreno et al., 2009; Yucel et al., 2009; Torppa et al., 2014). Further research with pre- and post-lingually deafened CI musicians and non-musicians, with or without music training provided, may reveal whether patterns developed during previous acoustic hearing or during post-implantation electric hearing may benefit pitch, music, and speech perception after implantation.

## Conclusions

In this study, performance of musicians and non-musicians was compared for a variety of speech and music listening tasks, with and without the spectro-temporal degradations associated with CI signal processing. Major findings include:

1. Cross-domain (music training to speech perception) effects were weak for speech intelligibility. The musician effect was minimal for word identification in noise, and non-existent for sentence identification in noise.
2. As pitch cues became more important for the listening task (i.e., vocal emotion identification, or MCI), the musician effect was more pronounced, suggesting that the musician effect may be rooted in better pitch perception.
3. Musicians tended to outperform non-musicians when listening to the CI simulation, especially for the MCI task. This suggests that musicians were better able to extract the relatively weak pitch and timbre information encoded in the CI simulations.

### Conflict of interest statement

The authors declare that the research was conducted in the absence of any commercial or financial relationships that could be construed as a potential conflict of interest.
